# Innovative use of biowaste based cementitious grouts for semi-flexible pavement application and optimization using response surface methodology

**DOI:** 10.1371/journal.pone.0335150

**Published:** 2025-10-23

**Authors:** Muhammad Imran Khan

**Affiliations:** Department of Civil Engineering, College of Engineering, Imam Mohammad Ibn Saud Islamic University (IMSIU), Riyadh, Saudi Arabia; Graphic Era Deemed to be University, INDIA

## Abstract

This study investigates the potential usage of biowaste (i.e., bagasse ash) in cementitious grouts for semi-flexible pavement applications. Cementitious grouts were prepared by partially replacing cement with bagasse ash (5%, 10%, 15% and 20%) and at w/c ratios of 0.30 to 0.40. Flow cone apparatus was used to determine the flow properties of fresh cementitious grouts. The hardened specimens of cementitious grouts also were tested for compressive strength at curing ages of 7 and 28 days. Moreover, response surface methodology (RSM) was used to analyze the relationships between independent/input variables (bagasse ash and w/c ratio) and dependent/output variables (flow and compressive strength). Compressive strength tests revealed that 7-day strength ranged from 27 MPa to 41 MPa, while 28-day strength ranged from 35 MPa to 65 MPa. Results indicate that bagasse ash significantly influences the flowability and compressive strength of the cementitious grouts, with optimal performance achieved at a 15% replacement level and a 0.35 w/c ratio. The optimal combination achieved a flow value of 16 seconds, a 7-day compressive strength of 32 MPa, and a 28-day compressive strength of 49 MPa. Response surface methodology (RSM) confirmed these results, identifying an optimized mix composition of 16% bagasse ash and a 0.35 w/c ratio. The findings demonstrate the potential of bagasse ash as a sustainable alternative to cement, contributing to reduced environmental impact and improved material performance in semi-flexible pavements.

## 1. Introduction

Semi-flexible pavement (SFP), also known as Grouted Macadam, is gaining recognition for its unique composition, which integrates an open-graded asphalt mixture with specialized cementitious grouting materials [1]. The hardened cementitious grouts interlocks with the asphalt mixture mastic (comprising fine aggregates, filler, and binder). This integration forms a single structure referred to as SFP, significantly enhancing its resistance to load-bearing, rutting, and deformation under pressure [2]. It is designed to offer improved durability, load-bearing capacity, and resistance to rutting failure while maintaining some flexibility [3–5]. SFP is a hybrid pavement type that has attracted considerable interest from researchers. It consists of cementitious grout materials that fill a high proportion of air voids (ranging from 25% to 35%) in porous asphalt [5–7]. Compared to conventional concrete and flexible pavements, SFP offers notable advantages, including excellent resistance to rutting and superior durability. It is considered a viable alternative pavement option that addresses the limitations of both concrete and flexible pavements [8]. According to Koting et al., SFP demonstrates strong performance against fatigue resistance, oil spills, and permanent deformation [9]. However, a significant limitation of SFP surfacing lies in the extensive requirement for cementitious grout, which increases construction costs. Additionally, the use of Portland cement in cementitious grouts formulations for SFP composites contributes to greenhouse gas emissions. Therefore, researchers are looking for utilizing waste material or by products to replace cement in the formulation of cementitious grouts for semi-flexible pavement application.

Research highlights the critical role of cement-based grouting materials in determining the performance of semi-flexible pavement (SFP) [10]. Studies indicate that cement-based grouts contribute significantly to the strength of semi-flexible pavement materials (SFPM), outperforming traditional matrix asphalt mixtures. The compressive strength of SFP closely aligns with that of the cementitious grouting material, suggesting that the latter governs the overall strength of SFPM [11–13]. A. Setyawan [14] further supports this, noting that the strength of SFPM is primarily influenced by the cement binder, with cold-mix grouted composites exhibiting lower compressive strength compared to hot-mix grouted materials. Additionally, Yang et al. [15] demonstrated that incorporating waste rubber powder enhances the low-temperature crack resistance of SFPM, though the degree of improvement varies with the injection method.

Wang et al. [16] found that adding flexible latex materials to cement-based grout improves SFPM performance, while Liu [17] observed that certain modified materials enhance temperature resistance but negatively impact high-temperature and water resistance. Furthermore, cement pastes in SFPM often include supplementary materials like fly ash, silica fume, ground granulated blast furnace slag, and marble powder as partial cement replacements.

Using industrial byproducts like bagasse ash to partially replace cement in semi-flexible pavement grouts offers clear sustainability and performance benefits, similar to marble powder (WMP) or fly ash in self-compacting concrete (SCC). Incorporating bagasse ash significantly reduces the carbon footprint and embodied energy while enhancing cementitious grout microstructure through filler effects and pozzolanic reactions. Studies indicate optimal replacement levels (typically 5–10% for cementitious materials) improve mechanical strength, chemical resistance (e.g., to acids/sulfates), and critical flow properties. Life cycle assessments confirm notable reductions in Global Warming Potential (GWP, 5–20%) and production costs [18–20]. Recent research also advances recycled aggregate concrete (RAC). Yu et al. (2024) used computational methods (Bayesian updating, Cuckoo search) to optimize precast RAC (PRAC) mixes balancing performance, cost, and sustainability. Concurrently, studies show that while recycled aggregates degrade ambient strength and post-peak behavior, their porosity reduces spalling at high temperatures (600–800°C) and can better preserve relative residual strength/modulus versus natural aggregates. Crucially, both areas highlight that recycled aggregate quality and substitution rates (typically 60–100%) are key to achieving sustainable concrete [21,22].

The environmental footprint of the concrete industry is greatly affected by the high cement consumption. Cement production alone accounts for a large portion of all industrial emissions, making cement a significant source of CO_2_ emissions [23]. In response to this problem, scientists have investigated the possibility of using agricultural ash as a partial replacement for cement in concrete mixes for the purpose of sustainable construction and environmental improvement. Extensive research has shown that the incorporation of agricultural waste ash into concrete can increase the durability of concrete, resist chloride and acid attacks, and also reduce its permeability. This improvement can be attributed to the high amorphous silica content in the ash formed during the combustion process. Amorphous silica promotes the formation of calcium silicate hydrate, a key component that contributes to the strength and function of concrete. There are many types of agricultural wastes available for this purpose, including straw ash, rice husk ash, palm paste ash, coconut ash, and sugar bagasse ash, each with specific properties and benefits for building construction [23–26].

Sugarcane bagasse is widely recognized as one of the most commonly utilized agricultural residues in the construction industry, primarily as a partial cement substitute in concrete applications [27]. Bagasse is a by-product generated during sugarcane processing in sugar mills, with approximately 25% of the sugarcane weight yielding bagasse for every kilogram processed [28]. When bagasse is combusted, it produces sugarcane bagasse ash, which is rich in pozzolanic oxides, including silica, alumina, and ferric oxide, collectively exceeding 70%. The pozzolanic activity of sugarcane bagasse ash is largely determined by the presence of amorphous silica, with its reactivity being strongly influenced by the surface area of the ash particles [29]. Following combustion, sugarcane bagasse ash is finely ground and incorporated into cement to create blended cement. Its properties are significantly shaped by factors such as the burning temperature and duration, cooling period, and grinding conditions. This composition highlights sugarcane bagasse ash potential as an effective pozzolanic material within cement-based systems [30]. Given its extensive use in construction, numerous studies have reviewed the incorporation of sugarcane bagasse ash as a supplementary cementitious material in concrete [31–33].

This study introduces the novel use of bagasse ash, a biowaste rich in amorphous silica, as a partial cement replacement in highly flowable cementitious grouts for semi-flexible pavement applications. While bagasse ash has been previously investigated as a pozzolanic material in concrete and mortar, its use in cementitious grouts for filling the interconnected voids of open-graded asphalt mixtures has not been reported in the literature. Unlike conventional concrete applications, the performance requirements cementitious grouts for semi-flexible pavement demand a unique balance of high flowability and adequate strength. In this work, cement was partially replaced with 5–20% bagasse ash and the water-to-cement ratio was varied between 0.30 and 0.40 to investigate their combined effects on cementitious grout’s flowability and compressive strength. Flow was measured using a flow cone apparatus, and compressive strength was determined on hardened cubes. Response Surface Methodology (RSM) was employed not only to statistically analyze the influence of these parameters but also to optimize the cementitious grout composition for the specific performance criteria of semi-flexible pavements.

## 2. Materials and methods

### 2.1. Materials and cementitious grouts preparation

**Fig 1:**
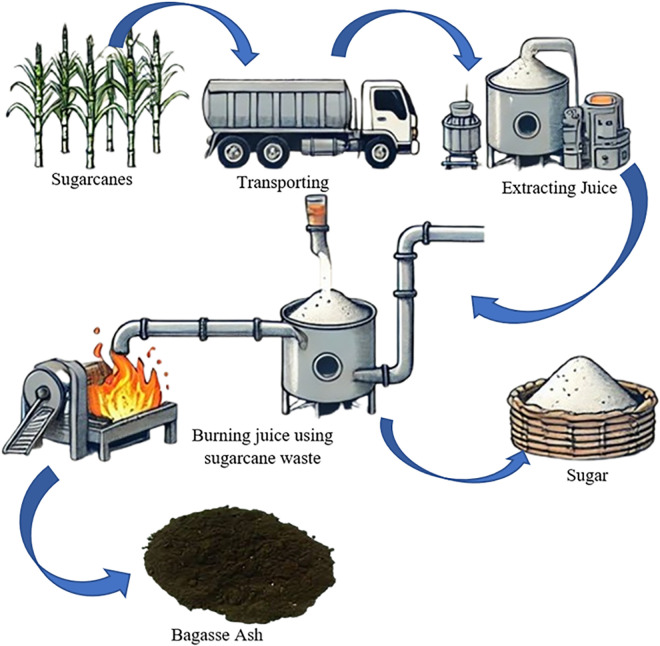
Production of Bagasse Ash.

The materials utilized in this study include ordinary Portland cement, bagasse ash, and a superplasticizer, all of which were sourced from local suppliers to ensure accessibility and consistency. The bagasse ash was obtained from sugar mills, where sugarcane is processed to extract juice. The residual sugarcane bagasse is used as fuel to heat the extracted juice during the production process. Once the combustion is complete, the resulting ash is collected, thoroughly dried in an oven to remove any moisture, and then sieved using a #200 sieve to achieve the desired particle size for the study. The Process is shown in Fig 1.

Ordinary Portland Cement (OPC), meeting ASTM C150 standards, was utilized as main binder in cementitious grouts. To enhance the flowability of the cementitious grouts, especially at low w/c ratios, a superplasticizer (Polycarboxylate Ether type) was utilized. This additive was selected for its ability to significantly improve workability while maintaining the cementitious grouts’ strength and durability. These properties were critical in achieving the desired flow characteristics for the cementitious grouting applications.

Various cementitious grouts were formulated by replacing Ordinary Portland Cement (OPC) with Bagasse Ash at proportions of 5%, 10%, 15%, and 20% by weight. The mixtures were prepared using w/c ratios of 0.30, 0.35, and 0.40. To improve flowability while maintaining strength, a superplasticizer (SP) was included at 0.5% of the binder’s weight. The cementitious grout preparation adhered to ASTM C305 [34] guidelines, utilizing a Hobart mixer to achieve consistent and uniform mixtures. The chemical composition of bagasse ash is shown in Table 1. The chemical composition of bagasse ash indicates its pozzolanic properties, primarily due to its high silica (SiO₂) content of 68.73%. Pozzolanic materials react with calcium hydroxide to form additional calcium silicate hydrate (C-S-H) gel, improving strength and durability.

**Table 1:** Chemical composition of bagasse ash.

Chemical Formula	Weight %
CaO	7.98
Na_2_O	1.45
SiO_2_	68.73
Al_2_O_3_	3.90
Fe_2_O_3_	7.86
MgO	1.76
Cl	0.45
K_2_O	7.87

### 2.2. Flow and compressive strength tests

Compressive strength and flow value were the two main criteria used to assess cementitious grout performance for grouted macadam applications. In compliance with ASTM C939 guidelines, a flow cone apparatus was used to measure the flow value [35]. 1725 milliliters of newly mixed cementitious grout were added to the flow cone for this test, and the amount of time it took for the cementitious grout to completely drain out was recorded in seconds. The cementitious grouts’ flow value (also known as their flow-out time) had to be between 11 and 16 seconds in order to be deemed appropriate for use [4,36,37]. For the cementitious grout to successfully fill in the spaces in open-graded asphalt mixtures without resulting in segregation or excessive bleeding, this flow range is necessary. Compressive strength was assessed as a crucial determinant of the mechanical performance of the hardened cementitious grout in addition to flowability. Compressive strength tests were performed on the specimens after seven and twenty-eight days of curing. A universal testing equipment with a 3000 kN capacity and a 1.350 kN/s loading rate was used to conduct these tests. For every cementitious grout modification, 50 mm x 50 mm x 50 mm cube specimens were made in accordance with ASTM C109 specifications for cement mortar specimens [38]. After 24 hours, the specimens were demolded and then cured in water at a regulated temperature until they reached the designated testing age. After that, the specimens underwent testing for compressive strength. In order to guarantee the precision and repeatability of the results, triplicate samples were made for each mix before the specimens were put through a compressive strength test.

### 2.3. Response surface methodology application

Response Surface Methodology (RSM) was employed to develop predictive models and investigate the interactive effects of the water-to-cement (w/c) ratio and Bagasse Ash (BA) content on the properties of cementitious grouts. Design Expert® software (Version X) was used for the experimental design, statistical analysis, and optimization. A custom design option was selected in RSM because the levels of the independent variables, w/c ratio and BA content, were pre-determined based on preliminary trials and practical considerations. The w/c ratio was fixed at three levels: 0.30, 0.35, and 0.40, while BA content was varied at four levels: 5%, 10%, 15%, and 20% by weight of cement. The factors and responses are summarized in Table 2. The w/c ratio (X_i_) and BA content (X_j_) served as the independent variables, while the measured responses included the flow value (Y_1_), 7-day compressive strength (Y_2_), and 28-day compressive strength (Y_3_). These responses were selected to represent both fresh (flow value) and hardened (compressive strengths) performance characteristics of the cementitious grouts. Flow value was measured in seconds (sec), and compressive strengths were recorded in megapascals (MPa).

**Table 2:** Detail of factors and responses.

	Factor		Responses		
	w/c	Bagasse Ash	Flow	7d CS	28d CS
Levels	0.30, 0.35, 0.40	5, 10, 15, 20	---	---	---
Code	X_i_	X_j_	Y_1_	Y_2_	Y_3_
Unit	---	%	sec	MPa	MPa

Experimental data obtained from laboratory tests were fitted to a second-order polynomial regression model, as given in Equation 1 [39], to establish the relationship between the independent variables and each response:


$$\begin{array}{c} Y=c+\sum a_{i}X_{i}+\sum a_{ii}{X_{i}}^{\text{2}}+\sum a_{ij}X_{i}X_{j}\; \end{array}$$ (1)

Here, *Y* represents the predicted response, *c* is the intercept, a_i_ is the linear coefficient, a_ii_ is the quadratic coefficient, and a_ij_ is the interaction coefficient for the respective factors.

The model adequacy and significance of each factor and their interactions were evaluated using Analysis of Variance (ANOVA). Statistical parameters, including the coefficient of determination (R²), adjusted R², predicted R², and p-values, were analyzed to assess the model’s predictive ability and fit quality. This systematic approach allowed for optimization of w/c ratio and BA content to achieve the desired balance between workability and strength, while also providing insights into the complex factor–response relationships.

RSM enabled a systematic exploration of optimal w/c ratios and Bagasse Ash content to achieve desired cementitious grout properties, enhancing efficiency and providing valuable insights into variable relationships.

## 3. Results and discussion

### 3.1. Flow values of cementitious grouts

**Fig 2:**
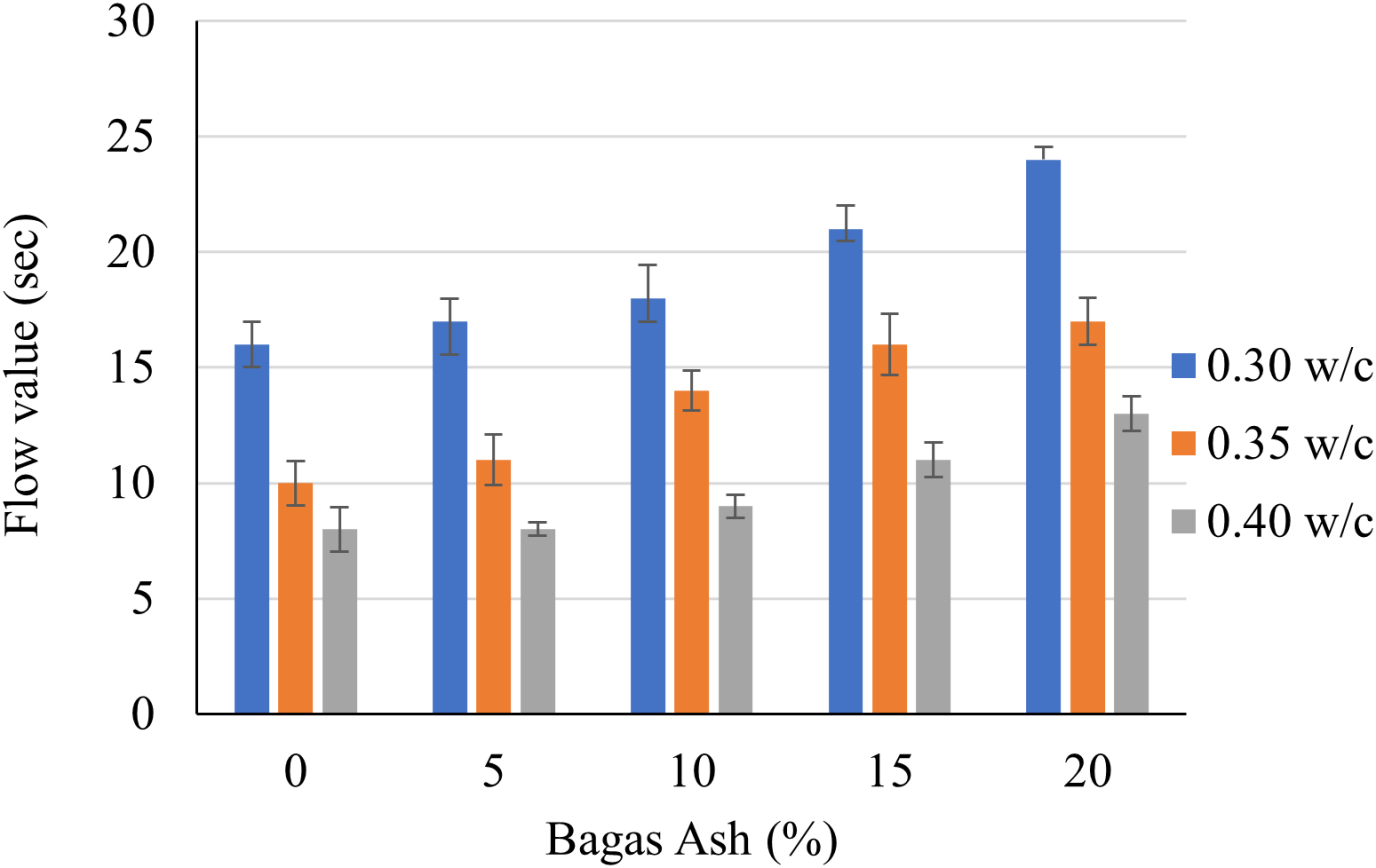
Flow value of cementitious grouts.

The Fig 2 demonstrates the relationship between flow values (in seconds), Bagasse Ash (BA) percentages (5%, 10%, 15%, and 20%), and w/c ratios (0.3, 0.35, and 0.4). It is evident that the flow values increase with a rise in Bagasse Ash percentage for all w/c ratios. Conversely, the flow values decrease as the w/c ratio increases for a given Bagasse Ash percentage. For a w/c ratio of 0.3, the flow value increases significantly from 17 seconds at 5% BA to 27 seconds at 20% BA, indicating a 58.8% rise. Similarly, at a 0.35 w/c ratio, the flow value rises from 12 seconds at 5% BA to 20 seconds at 20% BA, showing a 66.7% increase. For a w/c ratio of 0.4, the flow value grows from 10 seconds at 5% BA to 15 seconds at 20% BA, reflecting a 50% increase.

Examining the effect of the w/c ratio at a constant Bagasse Ash percentage, the flow values decrease as the w/c ratio increases. For instance, at 5% BA, the flow value decreases from 17 seconds at a w/c ratio of 0.3 to 12 seconds at 0.35 and further drops to 10 seconds at 0.4, resulting in an overall 29.4% decrease. A similar trend is observed at 20% BA, where the flow value reduces from 27 seconds at 0.3 to 20 seconds at 0.35 and further to 15 seconds at 0.4. These observations highlight the combined influence of Bagasse Ash content and w/c ratio on the flow behavior, with Bagasse Ash promoting higher flow values and increased w/c ratios reducing them. The flow decreases with increasing Bagasse Ash due to its higher fineness and angularity compared to cement, which increases the mix’s internal friction and reduces workability. With increasing w/c ratio, the flow increases because the additional water lubricates the particles, improving workability and reducing the resistance to flow.

### 3.2. Cementitious grout’s compressive strength

**Fig 3:**
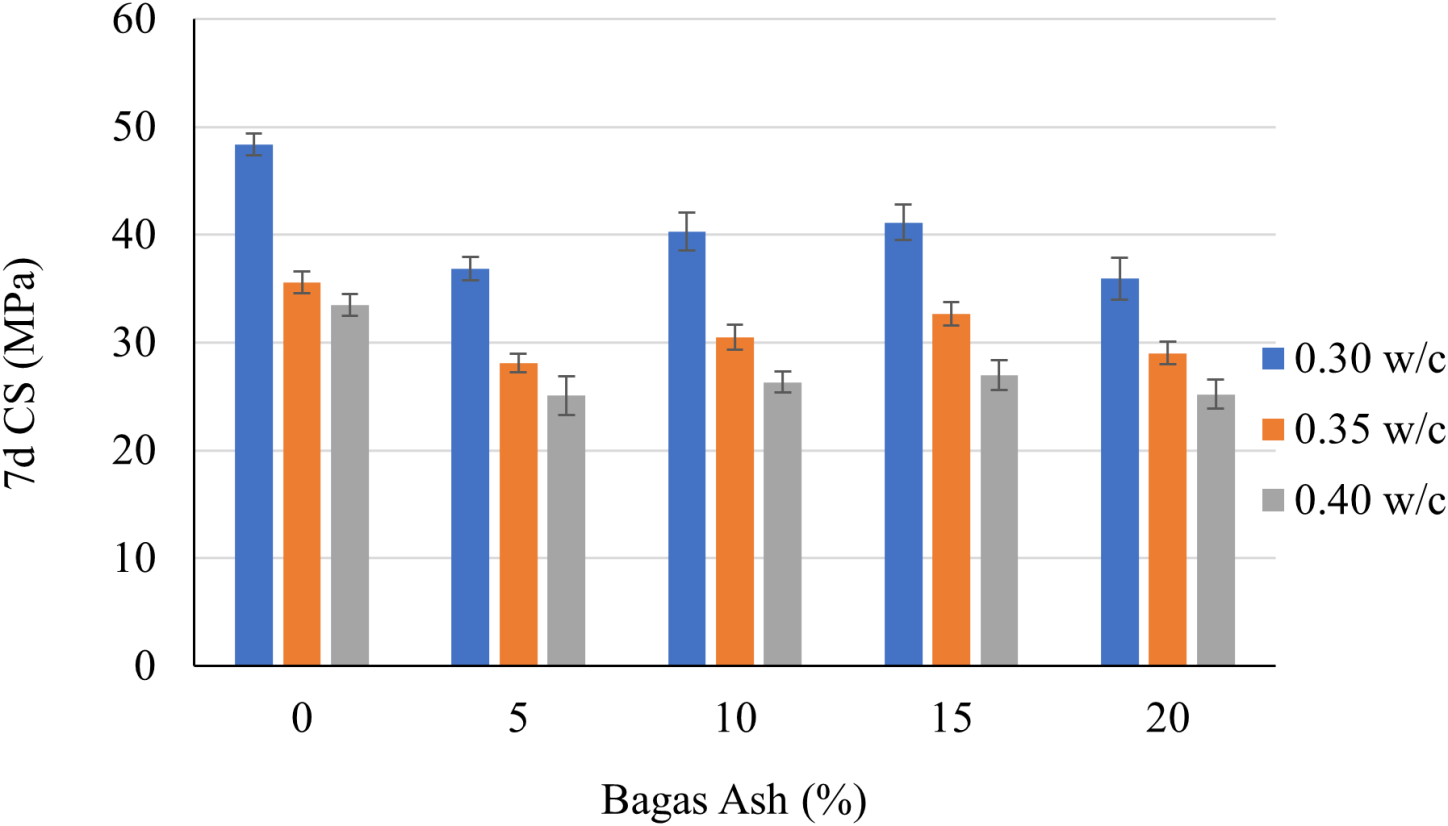
7d Compressive Strength of cementitious grouts.

**Fig 4:**
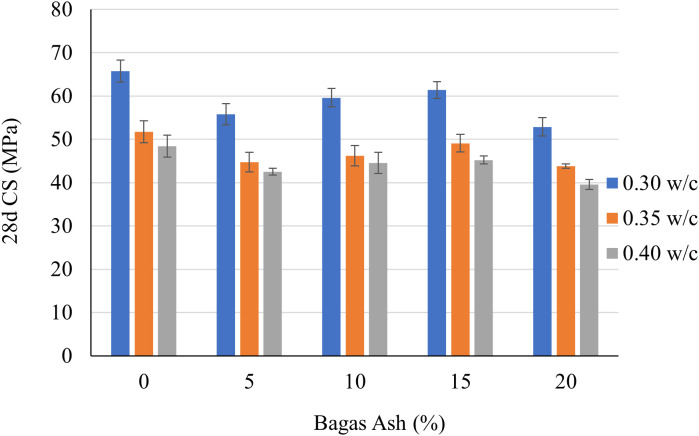
28d Compressive Strength of cementitious grouts.

The graphs (shown in Figs 3 and 4) illustrate the compressive strength of cementitious grouts at 7 days and 28 days, influenced by varying Bagasse Ash (BA) percentages (5%, 10%, 15%, and 20%) and w/c (0.3, 0.35, and 0.4). Compressive strength serves as an indicator of the material’s ability to withstand load-bearing pressures. The data reveals how the incorporation of Bagasse Ash and w/c ratio affect the mechanical performance of the cementitious grout over time.

For the 7-day compressive strength (Fig 3), it is evident that the w/c ratio has a significant impact. Across all Bagasse Ash percentages, the lowest w/c ratio (0.3) consistently delivers the highest compressive strength. For instance, at 5% Bagasse Ash, the 7-day strength reaches approximately 38 MPa for the 0.3 ratio, compared to around 32 MPa and 27 MPa for the 0.35 and 0.4 ratios, respectively. As the Bagasse Ash percentage increases, the 7-day compressive strength also improves, peaking at 15% Bagasse Ash with a compressive strength of about 41 MPa for the 0.3 ratio. However, further increasing Bagasse Ash to 20% results in a slight decline in strength, suggesting that higher ash content may hinder the hydration process at early curing stages. The variations in strength between the w/c ratios highlight the importance of minimizing water content to enhance early-age strength. The percentage differences between 0.3 and 0.4 ratios range from 25% to 30%, underscoring the influence of water content.

At 28 days, the compressive strength (as shown in Fig 4) increases for all combinations, reflecting continued hydration and the pozzolanic reaction of Bagasse Ash over time. Similar to the 7-day results, the 0.3 water-to-cement ratio produces the highest strength values, followed by 0.35 and 0.4. For 5% Bagasse Ash, the strength increases to approximately 60 MPa for the 0.3 ratio, while the 0.4 ratio lags behind at around 48 MPa. The optimal performance is again observed at 15% Bagasse Ash, where the compressive strength reaches nearly 65 MPa with the 0.3 ratio. Beyond 15% Bagasse Ash, the 28-day strength begins to decline, which suggests that excessive ash content may lead to a dilution effect, reducing the available cementitious material.

Overall, the data demonstrates that the combination of a 0.3 water-to-cement ratio and moderate Bagasse Ash content (around 15%) yields the highest compressive strength. Lower water-to-cement ratios promote denser microstructures and better hydration, while moderate ash content enhances the pozzolanic reaction, improving long-term strength. However, excessive water or ash content compromises strength by either increasing porosity or reducing active binder material. These findings emphasize the need for careful optimization of mix design to achieve desired performance.

### 3.3. Response surface methodology

#### 3.3.1. Model’s selection.

Table 3 from Design-Expert presents the fit summary and model selection for three responses: flow value, 7d CS, and 28d CS. The Table 3 evaluates different models (Linear, 2FI, Quadratic, and Cubic) based on several statistical parameters, including Sequential p-values, Lack of Fit p-values, Adjusted R², and Predicted R². For the Flow Value response, the Linear model is suggested as the best fit because it has a highly significant Sequential p-value (< 0.0001), an adequate Lack of Fit p-value (0.2533), and high Adjusted R² (0.8067) and Predicted R² (0.7827) values. These metrics indicate that the Linear model provides a robust and reliable fit for this response, while higher-order models such as Quadratic and Cubic either show aliasing or lower statistical significance.

**Table 3:** Fit Summary and model selection.

Source	Sequential p-value	Lack of Fit p-value	Adjusted R²	Predicted R²	Remarks
**Flow Value**					
Linear	< 0.0001	0.2533	0.8067	0.7827	Suggested
2FI	0.3814	0.2319	0.8054	0.7757	
Quadratic	0.9060	0.1261	0.7938	0.7461	
Cubic	0.0455	0.5172	0.8289	0.7625	Aliased
**7d Compressive Strength**					
Linear	< 0.0001	0.0069	0.7861	0.7615	
2FI	0.7676	0.0045	0.7800	0.7507	
Quadratic	< 0.0001	0.5525	0.8766	0.8488	Suggested
Cubic	0.1767	0.9667	0.8855	0.8455	Aliased
**28d Compressive Strength**					
Linear	< 0.0001	0.0002	0.6882	0.6534	
2FI	0.9335	< 0.0001	0.6785	0.6401	
Quadratic	< 0.0001	0.3457	0.8665	0.8363	Suggested
Cubic	0.1142	0.7843	0.8805	0.8360	Aliased

For the 7-day Compressive Strength, the Quadratic model is recommended as the best option due to its highly significant Sequential p-value (< 0.0001), acceptable Lack of Fit p-value (0.5525), and strong Adjusted R² (0.8766) and Predicted R² (0.8488) values. These results suggest that the Quadratic model provides excellent explanatory and predictive power for this response, outperforming the Linear model, which, although significant, has lower R² values. Similarly, for the 28-day Compressive Strength, the Quadratic model is also suggested as the best fit. It exhibits a highly significant Sequential p-value (< 0.0001), an adequate Lack of Fit p-value (0.3457), and high Adjusted R² (0.8665) and Predicted R² (0.8363), indicating strong predictive accuracy and reliability. The Cubic models for both compressive strength responses are aliased, and the Linear models have lower predictive capabilities, making the Quadratic model the most suitable choice. Overall, the Table 3 provides clear statistical justification for selecting the Linear model for Flow Value and the Quadratic model for the two compressive strength responses, ensuring accurate modeling and prediction of the experimental outcomes.

#### 3.3.2. ANOVA and fit statistics for the three responses.

The Table 4 provides the Analysis of Variance (ANOVA) results for three different responses: flow value (Linear model), 7d CS (Quadratic model), and 28d CS (Quadratic model). ANOVA is used to evaluate the significance of the factors and their interactions in the models based on statistical measures such as the sum of squares, mean square, F-value, and p-value.

**Table 4:** ANOVA for Responses.

Source	Sum of Squares	Mean Square	F-value	p-value		Variance Inflation Factors (VIFs)
**ANOVA for Linear model – (Response 1: Flow value)**						
Model	591.55	295.77	74.02	< 0.0001	significant	
A-Bagasse Ash	64.20	64.20	16.07	0.0003		1.00
B-w/c ratio	527.34	527.34	131.97	< 0.0001		1.00
Residual	131.86	4.00				
Lack of Fit	44.86	4.98	1.38	0.2533	not significant	
**ANOVA for Quadratic model – (Response 2: 7d-CS)**						
Model	1076.72	215.34	50.75	< 0.0001	significant	
A-Bagasse Ash	0.7987	0.7987	0.1882	0.6675		1.00
B-w/c ratio	960.39	960.39	226.32	< 0.0001		1.00
AB	0.6720	0.6720	0.1584	0.6935		1.00
A²	77.82	77.82	18.34	0.0002		1.00
B²	37.04	37.04	8.73	0.0060		1.00
Residual	127.30	4.24				
Lack of Fit	22.06	3.68	0.8385	0.5525	not significant	
**ANOVA for Quadratic model – (Response 3: 28d-CS)**						
Model	1587.30	317.46	46.42	< 0.0001	significant	
A-Bagasse Ash	11.57	11.57	1.69	0.2032		1.00
B-w/c ratio	1253.97	1253.97	183.35	< 0.0001		1.00
AB	0.1166	0.1166	0.0170	0.8970		1.00
A²	179.11	179.11	26.19	< 0.0001		1.00
B²	142.52	142.52	20.84	< 0.0001		1.00
Residual	205.18	6.84				
Lack of Fit	46.99	7.83	1.19	0.3457	not significant	

For the Flow Value (Response 1), a Linear model is used. The overall model is significant due to p-value < 0.0001, indicating that the independent variables, Bagasse Ash (A) and w/c ratio (B), effectively explain the variability in the Flow Value. Among the factors, Bagasse Ash has a significant effect with a p-value of 0.0003, and the w/c ratio has an even stronger influence with a p-value < 0.0001. The F-value for the w/c ratio is remarkably high (131.97), showing that it is the dominant factor affecting the Flow Value. The Lack of Fit test, with a p-value of 0.2533, is not significant, indicating that the Linear model provides an adequate fit to the data without systematic errors.

For the 7-day Compressive Strength (Response 2), a Quadratic model is employed, which is also significant, as p-value is less than 0.0001. The w/c ratio is the most significant factor, with a p-value < 0.0001 and a very high F-value of 226.32, demonstrating its critical role in influencing the 7-day compressive strength. While Bagasse Ash has a p-value of 0.6675, indicating it is not significant as a linear factor, the quadratic terms (A² and B²) are both significant, with p-values of 0.0002 and 0.0060, respectively. This highlights the nonlinear relationships in the model. The interaction term (AB) is not significant (p-value = 0.6935). The Lack of Fit test, with a p-value of 0.5525, confirms that the Quadratic model adequately fits the data.

For the 28-day Compressive Strength (Response 3), another Quadratic model is applied, which is also significant (p-value < 0.0001). Similar to the 7-day compressive strength, the w/c ratio is the most influential factor, with a p-value < 0.0001 and a very high F-value of 183.35. The quadratic terms (A² and B²) are significant, with p-values of < 0.0001 and < 0.0001, respectively, indicating the presence of nonlinear effects. However, the linear effect of Bagasse Ash (p-value = 0.2323) and the interaction term AB (p-value = 0.8970) are not significant. The Lack of Fit test, with a p-value of 0.3457, suggests that the model fits the data well. Moreover, the Variance Inflation Factors (VIFs), are equal to 1.00 for all responses, which is well below the threshold (VIF < 10), confirming no multicollinearity among the factors and their quadratic terms, demonstrating stability and statistical significance of the regression coefficients.

Overall, the ANOVA results validate the choice of models for each response, with the Linear model being appropriate for the Flow Value and Quadratic models for both compressive strengths. The results emphasize the critical role of the w/c ratio and highlight the presence of nonlinear effects, particularly for compressive strengths. These findings provide a robust statistical basis for understanding the impact of Bagasse Ash and w/c ratio on the responses. (For reference, refer to Design-Expert User Guides or similar statistical analysis references for detailed methodologies on ANOVA interpretation.)

The Table 5 summarizes the fit statistics for three responses: Flow Value, 7-day Compressive Strength (7d-CS), and 28-day Compressive Strength (28-CS), highlighting the model’s predictive accuracy and reliability. For Flow Value, the R² (0.8177) indicates that 81.77% of the variation is explained by the model, with adjusted R² (0.8067) accounting for model complexity, and predicted R² (0.7827) confirming good predictive ability. The adequate precision of 22.4560 suggests a strong signal-to-noise ratio, while the coefficient of variation (C.V.) of 13.19% indicates moderate variability relative to the mean (15.15). For 7d-CS, the model achieves a higher R² (0.8943), adjusted R² (0.8766), and predicted R² (0.8488), showing strong explanatory and predictive power. The adequate precision is 19.0550, with a low C.V. of 6.54%, reflecting high precision relative to the mean (31.50). Similarly, for 28-CS, the R² (0.8855), adjusted R² (0.8665), and predicted R² (0.8363) demonstrate excellent fit and prediction accuracy. The adequate precision (18.7273) and a very low C.V. of 5.36% indicate the model’s robustness, with minimal variability compared to the mean (48.78).

**Table 5:** Fit Statistics for Responses.

Description	Flow Value	7d-CS	28-CS
R²	0.8177	0.8943	0.8855
Adjusted R²	0.8067	0.8766	0.8665
Predicted R²	0.7827	0.8488	0.8363
Adeq Precision	22.4560	19.0550	18.7273
Std. Dev.	2.00	2.06	2.62
Mean	15.15	31.50	48.78
C.V. %	13.19	6.54	5.36

#### 3.3.3. Diagnostic and 3-D Plots for Flow, 7d and 28d Compressive strengths.

**Fig 5:**
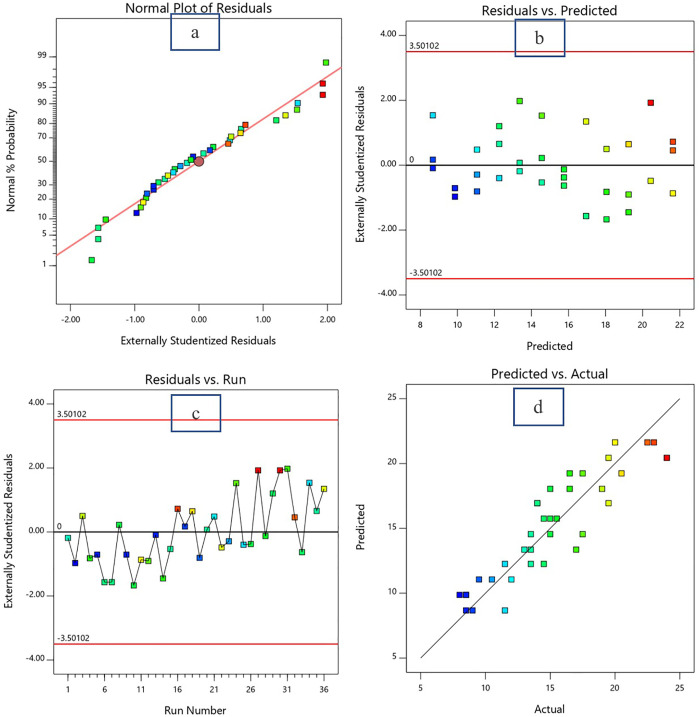
Diagnostic plots for Flow Value. a: Normal plot of residual.b: Residual vs predicted data plot. c: Residual vs run number plot.d: Predicted vs actual data plot.

**Fig 6:**
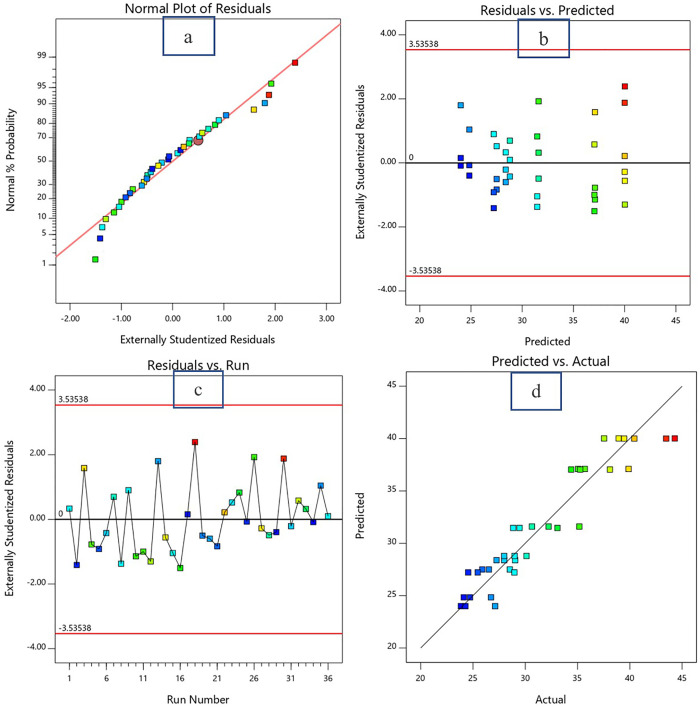
Diagnostic Plots for 7d CS. a: Normal plot of residual. b: Residual vs predicted data plot. c: Residual vs run number plot. d: Predicted vs actual data plot.

**Fig 7:**
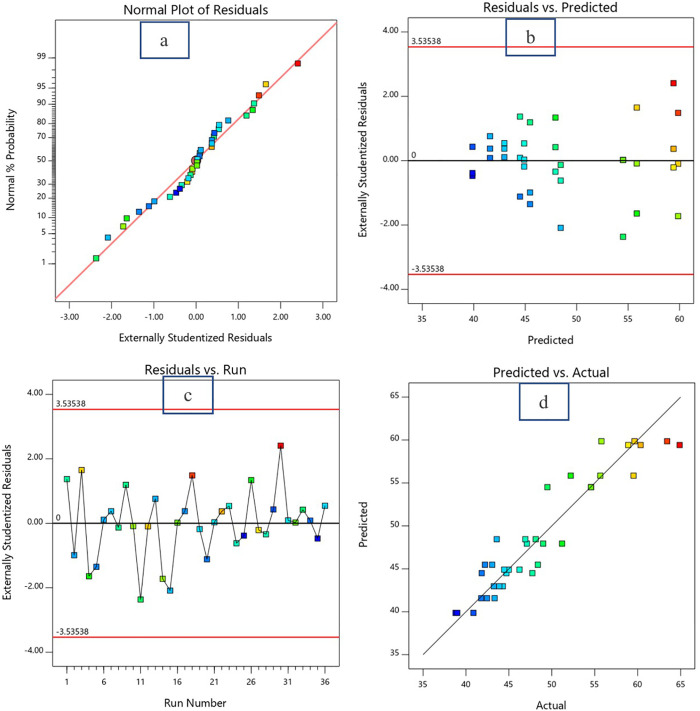
Diagnostic Plots for 28d CS. a: Normal plot of residual. b: Residual vs predicted data plot. c: Residual vs run number plotd: Predicted vs actual data plot.

The diagnostic plots presented in Figs 5-7 provide strong evidence of the statistical adequacy and reliability of the response surface methodology (RSM) model used to predict the responses (in this case flow value, 7d CS and 28d CS) as a function of the w/c ratio and bagasse ash content. The Normal Plot of Residuals (Figs 5a, 6a and 7a) demonstrates that the residuals are approximately normally distributed, as the data points align closely with the diagonal reference line for all responses. This alignment indicates that the assumption of normality is satisfied, which is crucial for the validity of statistical tests performed in the RSM analysis.

The Residuals vs. Predicted Plot (Figs 5b, 6b and 7b) confirms that the residuals are evenly scattered around the zero line without any discernible pattern. This random distribution indicates homoscedasticity, or constant variance of residuals across the range of predicted values. The residuals remain within the externally studentized limits of ±3.50102, which highlights the absence of influential outliers or extreme points that could compromise the model’s accuracy. Such behavior implies that the variability in the response (flow value) is consistently captured across the experimental domain, further validating the model’s reliability. In the Residuals vs. Run Number Plot (Figs 5c, 6c and 7c), the residuals fluctuate randomly around the zero line without showing any systematic trends. This result demonstrates that the experimental run order did not introduce bias or systematic errors, such as equipment drift or environmental influences, during the data collection process. The random distribution suggests that the residuals are independent and uncorrelated, satisfying another key assumption of RSM analysis. The Predicted vs. Actual Plot (Figs 5d, 6d and 7d) provides strong evidence of the model’s predictive accuracy. The predicted flow values are in close agreement with the actual experimental data, as indicated by the majority of the points lying near the diagonal line. The correlation coefficient (R²) for this relationship is likely high (close to 1.0), quantitatively supporting the model’s ability to explain the variability in the flow value. This alignment between predicted and actual values validates the model’s capability to accurately represent the relationship between the w/c ratio, bagasse ash, and flow value.

In summary, the diagnostic plots collectively confirm that the developed RSM model meets all statistical assumptions, including normality, independence, and homoscedasticity of residuals. The residuals remain within acceptable limits, and the model demonstrates a high level of predictive accuracy. These findings support the conclusion that the w/c ratio and bagasse ash are statistically significant factors influencing the flow value, and the model is well-suited for further optimization and practical application.

**Fig 8:**
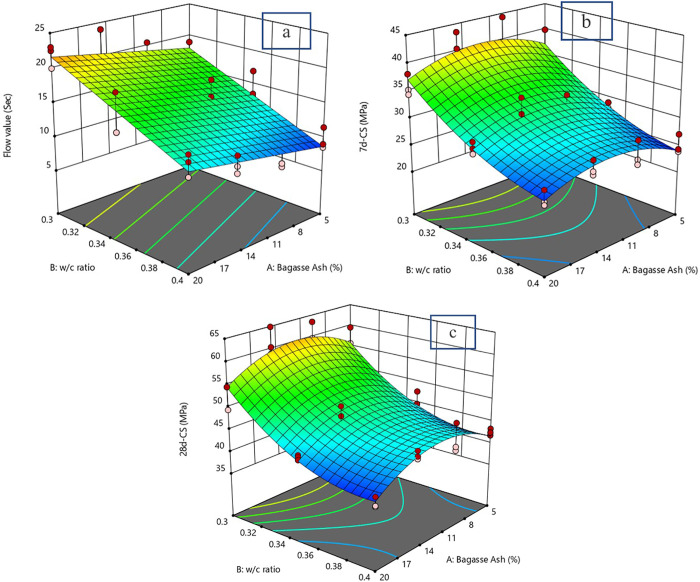
3D graph of flow and 7d and 28d compressive strength. a: 3D graph of flow value. b: 3D graph for 7d compressive strength. c: 3D graph for 28d compressive strength.

In addition to diagnostic plots, the effects of the w/c ratio and bagasse ash on flowability and compressive strength can also be illustrated using 3-dimensional plots, as shown in the Fig 8.

The Fig 8a illustrates the combined effects of Bagasse Ash content and the water/cement (w/c) ratio on the flow value of the mix, expressed in seconds. It is evident that the flow value increases significantly with the rise in Bagasse Ash content. At a low Bagasse Ash percentage (e.g., 5%), the flow value is relatively low, ranging between approximately 5–10 seconds depending on the w/c ratio. However, as the Bagasse Ash content increases to 20%, the flow value escalates, peaking at around 20–25 seconds. This behavior suggests that the inclusion of Bagasse Ash introduces a higher degree of resistance to flow, likely due to its pozzolanic and filler properties, which increase the viscosity of the mix. The w/c ratio, on the other hand, shows an inverse relationship with the flow value. At higher w/c ratios (e.g., 0.38 to 0.4), the flow value tends to decrease. For a given Bagasse Ash content, increasing the w/c ratio from 0.3 to 0.4 results in up to a 20% reduction in the flow value, reflecting a smoother and more workable mix at higher water contents. This indicates that while Bagasse Ash increases the stiffness of the mix, the w/c ratio counterbalances this effect by enhancing fluidity. The interplay between these two variables is crucial, as an optimum combination of Bagasse Ash and w/c ratio would provide a mix with desirable workability.

The Fig 8b examines the effect of Bagasse Ash content and w/c ratio on the 7-day compressive strength (CS), measured in MPa. Unlike the flow value, the 7-day CS exhibits a non-linear relationship with both parameters. At low Bagasse Ash content (e.g., 5%), the compressive strength is relatively high, with values ranging between 35 and 40 MPa, depending on the w/c ratio. However, as the Bagasse Ash percentage increases, the 7-day CS starts to decline. At 20% Bagasse Ash, the compressive strength reduces significantly, falling to values as low as 20–25 MPa. This reduction can be attributed to the dilution effect of Bagasse Ash, which replaces a portion of the cement and consequently lowers the early strength gain. The w/c ratio also influences the compressive strength. Lower w/c ratios (e.g., 0.3) correspond to higher compressive strength values, while higher w/c ratios (e.g., 0.38 to 0.4) lead to a noticeable decline in strength. For instance, at a Bagasse Ash content of 5%, reducing the w/c ratio from 0.4 to 0.3 results in an approximate 10% increase in compressive strength. This trend highlights the role of the w/c ratio in controlling the porosity of the mix, with lower ratios yielding denser and stronger matrices.

The graph (Fig 8c) illustrates the relationship between Bagasse Ash content, the water/cement (w/c) ratio, and the 28-day compressive strength (CS), measured in MPa. It provides insights into the long-term strength development of the mix and the interactions between these two factors. The 28-day compressive strength shows a more pronounced variation with Bagasse Ash content compared to the 7-day CS. At lower Bagasse Ash levels (e.g., 5%), the compressive strength is significantly higher, reaching values in the range of 55–65 MPa. This high strength is attributed to the optimal hydration process and minimal replacement of cement, which ensures a strong and dense matrix. As the Bagasse Ash content increases to higher percentages (e.g., 20%), a marked reduction in compressive strength is observed, with values dropping to around 35–40 MPa. This decline is likely due to the dilution effect of excessive cement replacement, reducing the availability of cementitious materials needed for strength development. Similar behaviors were reported by several researchers when bagasse ash was used as a cement replacement in concrete applications. The compressive strength of cementitious materials is influenced by the use of sugarcane bagasse ash in varying ways depending on the processing method and replacement percentage. Torres de Sande et al. (2022) found that grinding SCBA significantly enhances compressive strength, with up to a 62% increase at 28 days using 20% replacement due to improved pozzolanic reactivity and pore refinement [40]. Hussien and Qan (2022) reported that a 5% SCBA replacement yielded optimal strength gains in both mortar and concrete, while higher percentages led to reductions [41]. Gudia et al. (2023) observed that replacements up to 10% maintained or slightly improved strength, but further increases reduced compressive strength due to higher water demand and dilution effects [42]. Gupta et al. (2022), in their review, noted that strength generally increases up to 10% SCBA replacement, beyond which it declines due to insufficient free lime for pozzolanic reactions [43]. Overall, the optimal bagasse ash replacement level ranges between 5–15%, with processed ash (e.g., ground or sieved) yielding better results.

The w/c ratio also plays a vital role in the 28-day compressive strength. Lower w/c ratios (e.g., 0.3) consistently yield higher compressive strengths across all Bagasse Ash percentages, with strength values peaking at approximately 60–65 MPa for mixes with low Bagasse Ash content. This result reflects the reduced porosity and denser structure achieved with lower water content. On the other hand, increasing the w/c ratio to 0.4 leads to a significant decline in compressive strength, particularly at higher Bagasse Ash levels, where values can fall below 40 MPa. This trend indicates that higher water content increases porosity and weakens the matrix.

Interestingly, the graph highlights a critical balance between Bagasse Ash content and the w/c ratio. While lower Bagasse Ash content combined with a low w/c ratio maximizes compressive strength, increasing either parameter compromises the long-term strength. The contour lines at the base of the graph suggest an optimum range of Bagasse Ash (around 5–10%) and a w/c ratio of approximately 0.3–0.35 for achieving the best 28-day compressive strength performance. Overall, the graph underscores the trade-off between sustainability and performance. While incorporating Bagasse Ash enhances environmental benefits, excessive replacement levels negatively impact the strength properties, especially under higher w/c ratios. An optimized combination of these parameters is necessary to ensure both durability and sustainability in the mix design.

#### 3.3.4. Prediction equation and optimization.

**Fig 9:**
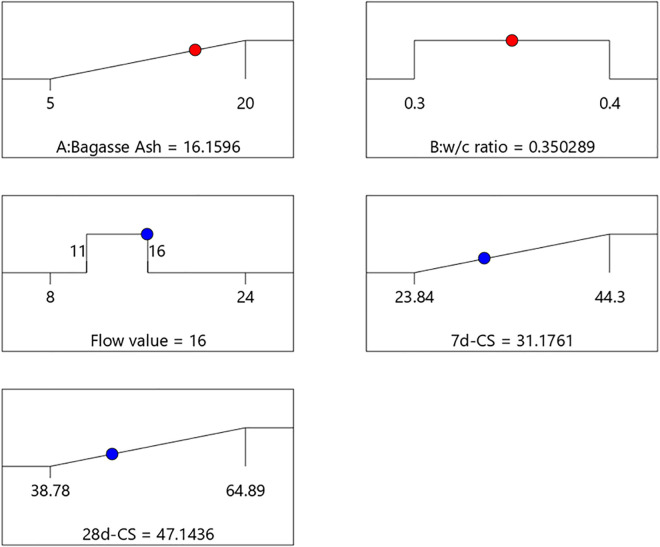
Optimization of cementitious grouts composition using RSM.


Linear and quadratic polynomial equations can be used to predict the responses. Equations 2–4 presents established polynomial equations, which can be used to establish models for predicting flowability and compressive strength. The Equations (2–4) 6 and Fig 9 present the predictive modeling and optimization results for flowability and compressive strength of cementitious grouts containing bagasse ash.

$$\begin{array}{c} Flow\;value=+\text{1}\text{5}.\text{1}\text{5}+\text{1}.\text{7}\text{9}A-\text{4}.\text{6}\text{9}B \end{array}$$ (2)

$$\begin{array}{c} \text{7}d\;CS=+\text{3}\text{1}.\text{9}\text{0}+\text{0}.\text{1}\text{9}A-\text{6}.\text{3}\text{3}B+\text{0}.\text{2}\text{2}AB-\text{3}.\text{3}\text{1}A^{\text{2}}+\text{2}.\text{1}\text{5}B^{\text{2}} \end{array}$$ (3)

$$\begin{array}{c} \text{2}\text{8}d\;CS=+\text{4}\text{8}.\text{7}\text{5}-\text{0}.\text{7}\text{6}A-\text{7}.\text{2}\text{3}B-\text{0}.\text{0}\text{9}AB-\text{5}.\text{0}\text{2}A^{\text{2}}+\text{4}.\text{2}\text{2}B^{\text{2}} \end{array}$$ (4)

Equations 2–4 are the developed linear and quadratic polynomial equations used to predict flow value, 7-day compressive strength (7d-CS), and 28-day compressive strength (28d-CS). The results indicate that the water-to-cement (w/c) ratio and bagasse ash content significantly influence the responses, with quadratic terms further refining the predictions for compressive strength.

Fig 9 illustrate the optimization process using response surface methodology (RSM). They show the effects of bagasse ash and w/c ratio on flow value and compressive strength, highlighting the optimized conditions: a bagasse ash content of 16.1596%, a w/c ratio of 0.350289, a flow value of 16, a 7-day compressive strength of 31.1761 MPa, and a 28-day compressive strength of 47.1436 MPa. These results confirm the utility of RSM in optimizing cementitious grout formulations to meet specific performance criteria.

## 4. Conclusions

This study assesses the potential of using biowaste (bagasse ash) as a partial replacement for cement in cementitious grouts intended for semi-flexible pavement applications. The cementitious grouts were analyzed for flow properties and compressive strength at 7 and 28 days. Additionally, statistical analyses were conducted to establish relationships between various factors and their corresponding responses. The following conclusions are drawn from the study.

- Flow values increased with bagasse ash content, ranging from 10 seconds at 5% bagasse ash to 27 seconds at 20% bagasse ash. Higher w/c ratios reduced flow resistance, with a maximum flow value of 15 seconds observed at a 0.4 w/c ratio and 20% bagasse ash.
- The 7-day compressive strength ranged from 27 MPa (20% bagasse ash, 0.40 w/c) to 41 MPa (15% bagasse ash, 0.30 w/c).
- The 28-day compressive strength peaked at 65 MPa with 15% bagasse ash and 0.30 w/c, but dropped to 35 MPa with 20% bagasse ash and 0.40 w/c.
- RSM models confirmed an optimal mix composition of 16% bagasse ash and 0.35 w/c ratio.
- Predicted values for the optimal mix were a flow time of 16 seconds, 7-day strength of 31 MPa, and 28-day strength of 47 MPa.
- The quadratic model for compressive strength demonstrated high predictive accuracy, with adjusted R² values of 0.8766 for 7-day strength and 0.8665 for 28-day strength.
- Bagasse ash effectively replaces up to 15% of cement in cementitious grout formulations, reducing greenhouse gas emissions and construction costs while maintaining performance.
- Excessive BA content (above 20%) diminishes early and long-term strength due to the dilution effect.
- The study highlights the potential of utilizing bagasse ash as a sustainable solution in cementitious grouts, reducing reliance on Portland cement and addressing environmental concerns.

### Future recommendation

Future studies should incorporate a detailed Life Cycle Assessment (LCA) to examine the overall sustainability of using bagasse ash as a cement replacement in cement grouts for semi-flexible pavements. This will help assess potential trade-offs between mechanical strength, economic feasibility, and environmental benefits, thereby ensuring a balanced evaluation of performance and sustainability.

## Supporting information

S1 Data Some data. (JPG)

## References

[1] Bharath G, Shukla M, Nagabushana MN, Chandra S, Shaw A. Laboratory and field evaluation of cement grouted bituminous mixes. Road Materials and Pavement Design. 2019;21(6):1694–712. doi: 10.1080/14680629.2019.1567375

[2] Taghipoor M, Hassani A, Karimi MM. Investigation of material composition, design, and performance of open-graded asphalt mixtures for semi-flexible pavement: A comprehensive experimental study. Journal of Traffic and Transportation Engineering (English Edition). 2024;11(1):92–116. doi: 10.1016/j.jtte.2023.06.003

[3] Al-Nawasir R, Al-Humeidawi B, Shubbar A. Influence of Sustainable Grout Material on the Moisture Damage of Semi-flexible Pavement. Period Polytech Civil Eng. 2024;68(3):961–73. doi: 10.3311/ppci.23373

[4] Al-Nawasir R, Al-Humeidawi B, Khan MI, Khahro SH, Memon ZA. Effect of glass waste powder and date palm seed ash based sustainable cementitious grouts on the performance of semi-flexible pavement. Case Studies in Construction Materials. 2024;21:e03453. doi: 10.1016/j.cscm.2024.e03453

[5] Ali R, Al-Humeidawi B. A scientometric study and a bibliometric review of the literature on the design and construction of semi-flexible pavement. QJES. 2023;16(2):82–91. doi: 10.30772/qjes.v16i2.921

[6] Khan MI, Sutanto MH, Khahro SH, Zoorob SE, Md. Yusoff NI, Al-Sabaeei AM, et al. Fatigue Prediction Model and Stiffness Modulus for Semi-Flexible Pavement Surfacing Using Irradiated Waste Polyethylene Terephthalate-Based Cement Grouts. Coatings. 2022;13(1):76. doi: 10.3390/coatings13010076

[7] Al-Nawasir R, Al-Humeidawi B, Shubbar A. Influence of Sustainable Grout Material on the Moisture Damage of Semi-flexible Pavement. Period Polytech Civil Eng. 2024;68(3):961–73. doi: 10.3311/ppci.23373

[8] Imran Khan M, Sutanto MH, Napiah MB, Zoorob SE, Al-Sabaeei AM, Rafiq W, et al. Investigating the mechanical properties and fuel spillage resistance of semi-flexible pavement surfacing containing irradiated waste PET based grouts. Construction and Building Materials. 2021;304:124641. doi: 10.1016/j.conbuildmat.2021.124641

[9] Koting S, Karim MR, Mahmud H, Mashaan NS, Ibrahim MR, Katman H, et al. Effects of using silica fume and polycarboxylate-type superplasticizer on physical properties of cementitious grout mixtures for semiflexible pavement surfacing. ScientificWorldJournal. 2014;2014:596364. doi: 10.1155/2014/596364 24526911 PMC3910231

[10] Ren J, Xu Y, Zhao Z, Chen J, Cheng Y, Huang J, et al. Fatigue prediction of semi-flexible composite mixture based on damage evolution. Construction and Building Materials. 2022;318:126004. doi: 10.1016/j.conbuildmat.2021.126004

[11] Zhang J, Cai J, Pei J, Li R, Chen X. Formulation and performance comparison of grouting materials for semi-flexible pavement. Construction and Building Materials. 2016;115:582–92. doi: 10.1016/j.conbuildmat.2016.04.062

[12] Cai X, Huang W, Wu K. Study of the Self-Healing Performance of Semi-Flexible Pavement Materials Grouted with Engineered Cementitious Composites Mortar based on a Non-Standard Test. Materials (Basel). 2019;12(21):3488. doi: 10.3390/ma12213488 31653082 PMC6862351

[13] Cai X, Shi C, Chen X, Yang J. Identification of damage mechanisms during splitting test on SFP at different temperatures based on acoustic emission. Construction and Building Materials. 2021;270:121391. doi: 10.1016/j.conbuildmat.2020.121391

[14] Setyawan A. Asessing the Compressive Strength Properties of Semi-Flexible Pavements. Procedia Engineering. 2013;54:863–74. doi: 10.1016/j.proeng.2013.03.079

[15] Yang Y, Ding Q, Huang C, Huang S. Research on the low-temperature cracking resistance of semi-flexible pavement with waste rubber powder. In: Proceedings of the Asphalt Rubber 2009 Conference. Nanjing, China, 2009.

[16] Wang D, Liang X, Jiang C, Pan Y. Impact analysis of Carboxyl Latex on the performance of semi-flexible pavement using warm-mix technology. Construction and Building Materials. 2018;179:566–75. doi: 10.1016/j.conbuildmat.2018.05.173

[17] Liu B, Liang D. Effect of mass ratio of asphalt to cement on the properties of cement modified asphalt emulsion mortar. Construction and Building Materials. 2017;134:39–43. doi: 10.1016/j.conbuildmat.2016.12.137

[18] Khataei B, Ahmadi M, Kioumarsi M, editors. Environmental Assessment of Fiber-Reinforced Self-Compacting Concrete Containing Class-F Fly Ash. The 1st International Conference on Net-Zero Built Environment; 2025 2025//; Cham: Springer Nature Switzerland.

[19] Ahmadi M, Abdollahzadeh E, Kioumarsi M. Using marble waste as a partial aggregate replacement in the development of sustainable self-compacting concrete. Materials Today: Proceedings. 2023. doi: 10.1016/j.matpr.2023.04.103

[20] Ahmadi M, Abdollahzadeh E, Kashfi M, Khataei B, Razavi M. Life Cycle Assessment and Performance Evaluation of Self-Compacting Concrete Incorporating Waste Marble Powder and Aggregates. Materials (Basel). 2025;18(13):2982. doi: 10.3390/ma18132982 40649470 PMC12250673

[21] Lin L, Xu J, Ying W, Yu Y, Zhou L. Post-fire compressive mechanical behaviors of concrete incorporating coarse and fine recycled aggregates. Construction and Building Materials. 2025;461:139948. doi: 10.1016/j.conbuildmat.2025.139948

[22] Yu Y, Fang G-H, Kurda R, Sabuj AR, Zhao X-Y. An agile, intelligent and scalable framework for mix design optimization of green concrete incorporating recycled aggregates from precast rejects. Case Studies in Construction Materials. 2024;20:e03156. doi: 10.1016/j.cscm.2024.e03156

[23] Marzouk HA, Arab MA, Fattouh MS, Hamouda AS. Effect of Agricultural Phragmites, Rice Straw, Rice Husk, and Sugarcane Bagasse Ashes on the Properties and Microstructure of High-Strength Self-Compacted Self-Curing Concrete. Buildings. 2023;13(9):2394. doi: 10.3390/buildings13092394

[24] He J, Kawasaki S, Achal V. The Utilization of Agricultural Waste as Agro-Cement in Concrete: A Review. Sustainability. 2020;12(17):6971. doi: 10.3390/su12176971

[25] Kamaruddin S, Goh WI, Abdul Mutalib NAN, Jhatial AA, Mohamad N, Rahman AF. Effect of Combined Supplementary Cementitious Materials on the Fresh and Mechanical Properties of Eco-Efficient Self-Compacting Concrete. Arab J Sci Eng. 2021;46(11):10953–73. doi: 10.1007/s13369-021-05656-x

[26] Prasittisopin L, Trejo D. Performance Characteristics of Blended Cementitious Systems Incorporating Chemically Transformed Rice Husk Ash. Advances in Civil Engineering Materials. 2017;6(1):17–36. doi: 10.1520/acem20160001

[27] Embong R, Shafiq N, Kusbiantoro A, Nuruddin MF. Effectiveness of low-concentration acid and solar drying as pre-treatment features for producing pozzolanic sugarcane bagasse ash. Journal of Cleaner Production. 2016;112:953–62. doi: 10.1016/j.jclepro.2015.09.066

[28] Souza AE, Teixeira SR, Santos GTA, Costa FB, Longo E. Reuse of sugarcane bagasse ash (SCBA) to produce ceramic materials. J Environ Manage. 2011;92(10):2774–80. doi: 10.1016/j.jenvman.2011.06.020 21733619

[29] Ahmed MM, Sadoon A, Bassuoni MT, Ghazy A. Utilizing agricultural residues from hot and cold climates as sustainable SCMs for low-carbon concrete. Sustainability. 2024;16(23).

[30] Arenas-Piedrahita JC, Montes-García P, Mendoza-Rangel JM, López Calvo HZ, Valdez-Tamez PL, Martínez-Reyes J. Mechanical and durability properties of mortars prepared with untreated sugarcane bagasse ash and untreated fly ash. Construction and Building Materials. 2016;105:69–81. doi: 10.1016/j.conbuildmat.2015.12.047

[31] Thomas BS, Yang J, Bahurudeen A, Abdalla JA, Hawileh RA, Hamada HM, et al. Sugarcane bagasse ash as supplementary cementitious material in concrete – a review. Materials Today Sustainability. 2021;15:100086. doi: 10.1016/j.mtsust.2021.100086

[32] Tripathy A, Acharya PK. Characterization of bagasse ash and its sustainable use in concrete as a supplementary binder – A review. Construction and Building Materials. 2022;322:126391. doi: 10.1016/j.conbuildmat.2022.126391

[33] Li Y, Chai J, Wang R, Zhang X, Si Z. Utilization of sugarcane bagasse ash (SCBA) in construction technology: A state-of-the-art review. Journal of Building Engineering. 2022;56:104774. doi: 10.1016/j.jobe.2022.104774

[34] ASTM 305, Standard practice for mechanical mixing of hydraulic cement pastes and mortars of plastic consistency. ASTM; 1999.

[35] ASTM. ASTM C939/C939M-2002 Standard Test Method for Flow of Grout for Preplaced Aggregate Concrete (Flow Cone Method). 2002.

[36] Khan MI, Sutanto MH, Napiah MB, Khan K, Rafiq W. Design optimization and statistical modeling of cementitious grout containing irradiated plastic waste and silica fume using response surface methodology. Construction and Building Materials. 2021;271:121504. doi: 10.1016/j.conbuildmat.2020.121504

[37] Khan MI, Sutanto MH, Napiah MB, Zoorob SE, Yusoff NIM, Usman A, et al. Irradiated polyethylene terephthalate and fly ash based grouts for semi-flexible pavement: design and optimisation using response surface methodology. International Journal of Pavement Engineering. 2020;23(8):2515–30. doi: 10.1080/10298436.2020.1861446

[38] ASTM. ASTM C109: Standard Test Method for Compressive Strength of Hydraulic Cement Mortars (Using 2-in. or [50-mm] Cube Specimens). ASTM International; 2013.

[39] Khan MI. Robust prediction models for flow and compressive strength of sustainable cement grouts for grouted macadam pavement using RSM. Construction and Building Materials. 2024;448:138205. doi: 10.1016/j.conbuildmat.2024.138205

[40] Torres de Sande V, Sadique M, Bras A, Pineda P. Activated sugarcane bagasse ash as efficient admixture in cement-based mortars: Mechanical and durability improvements. Journal of Building Engineering. 2022;59:105082. doi: 10.1016/j.jobe.2022.105082

[41] Hussien NT, Oan AF. The use of sugarcane wastes in concrete. J Eng Appl Sci. 2022;69(1). doi: 10.1186/s44147-022-00076-6

[42] Gudia SEL, Go AW, Giduquio MB, Juanir RG, Jamora JB, Gunarto C, et al. Sugarcane bagasse ash as a partial replacement for cement in paste and mortar formulation – A case in the Philippines. Journal of Building Engineering. 2023;76:107221. doi: 10.1016/j.jobe.2023.107221

[43] Gupta CK, Sachan AK, Kumar R. Utilization of sugarcane bagasse ash in mortar and concrete: A review. Materials Today: Proceedings. 2022;65:798–807. doi: 10.1016/j.matpr.2022.03.304

