# Supplementary figures and images for: Innovative use of biowaste based cementitious grouts for semi-flexible pavement application and optimization using response surface methodology
Source: PLoS One. 2025 Oct 23;20(10):e0335150. doi: 10.1371/journal.pone.0335150 (PMC12548891; doi:10.1371/journal.pone.0335150)

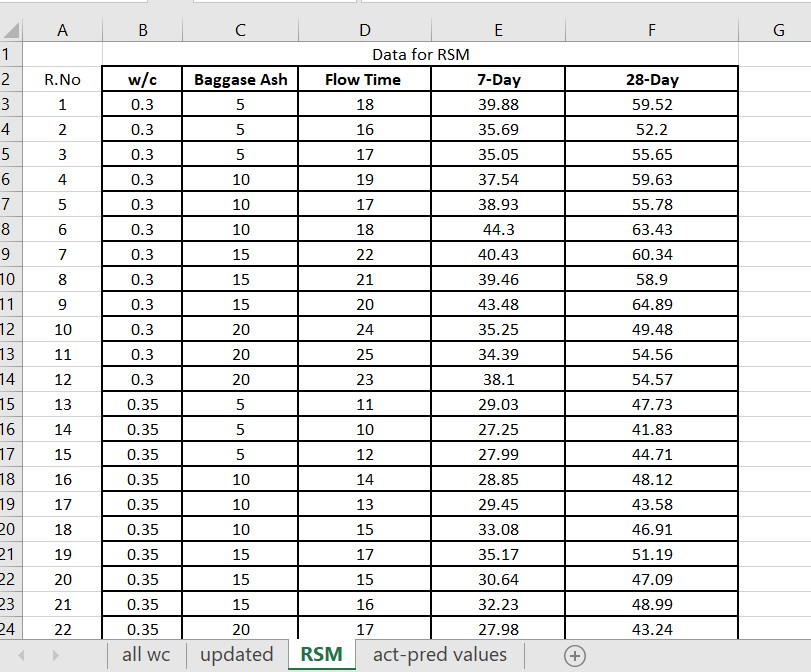

Supplement: S1 Data — (JPG) [file pone.0335150.s001.jpg]
